# Supplementary figures and images for: Improvement of muscle strength in a mouse model for congenital myopathy treated with HDAC and DNA methyltransferase inhibitors
Source: eLife. 2022 Mar 3;11:e73718. doi: 10.7554/eLife.73718 (PMC8956288; doi:10.7554/eLife.73718)

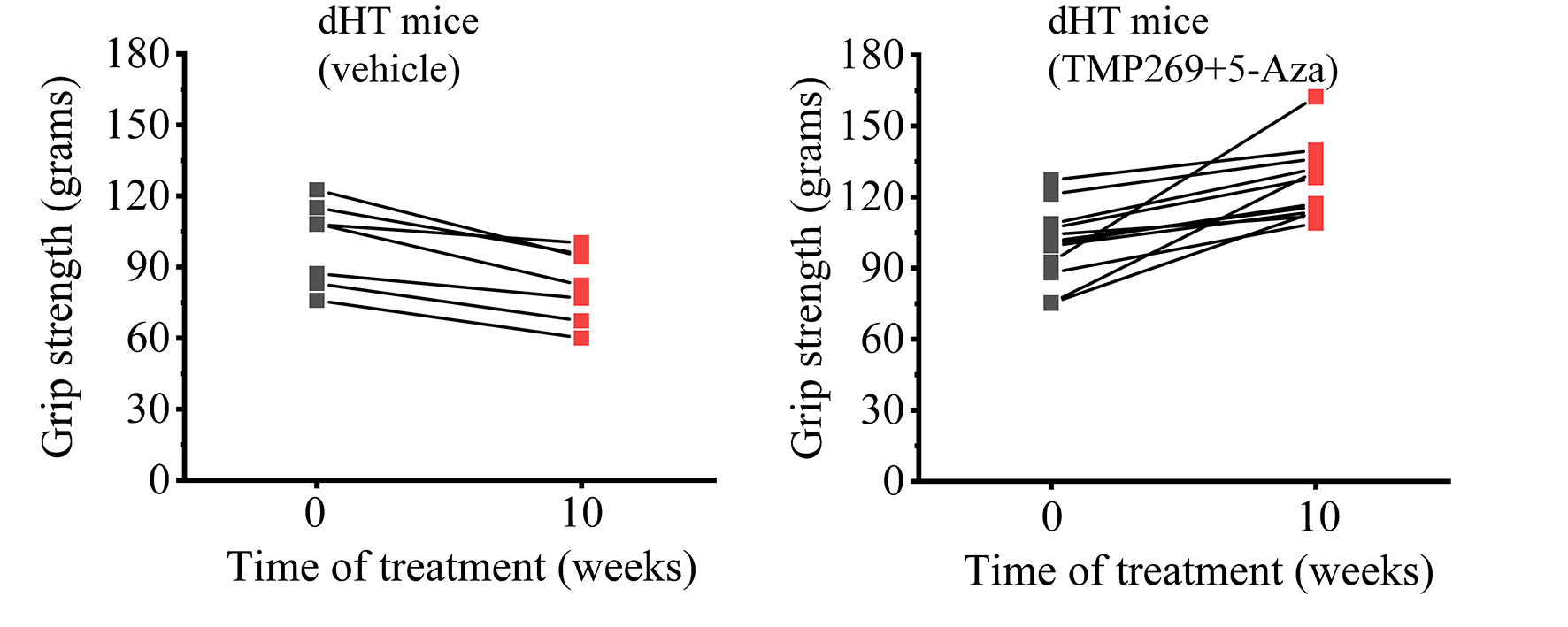

Supplement: Figure 1—source data 1. — Each line represents the average grip strength (in grams) per mouse, calculated by averaging five measurements obtained from the same mouse. The graphs show raw data of grip strength before treatment (t = 0; gray symbols) and 10 weeks after treatment (red symbols). N = 7 for vehicle-treated dHT mice and N = 12 TMP289 + 5-Aza-treated dHT mice. [file elife-73718-fig1-data1.zip › Figure 1A source file dHT treatment.tif]

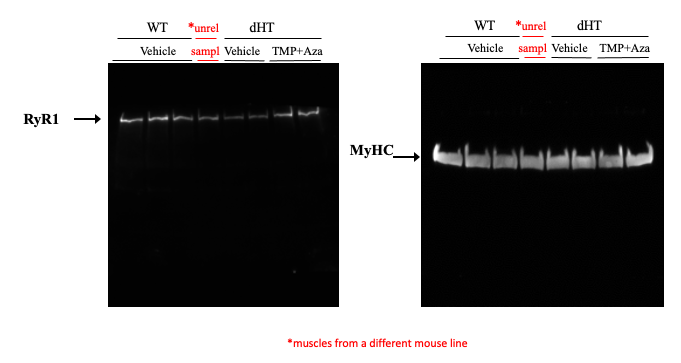

Supplement: Figure 5—source data 1. — Western blot analysis of RyR1 content in total homogenates of soleus muscle from wild type (WT) and dHT mice treated with vehicle or TMP269 + 5-Aza. Proteins were separated on a 6% SDS-PAGE, blotted overnight onto nitrocellulose. Left panel: images of blot probed with anti-RyR1 Ab used in Figure 5B. Right panel: images of blot probed with anti-MyHC Ab for normalization. WT Vehicle: wild type mouse treated with vehicle; dHT Vehicle: dHT mouse treated with vehicle; dHT TMP + Aza; dHT mouse treated with TMP269 + 5-Aza; unrel sampl: unrelated sample (sample belonging to a mouse line of a different project). [file elife-73718-fig5-data1.zip › Figure 5B-source data 1.tif]

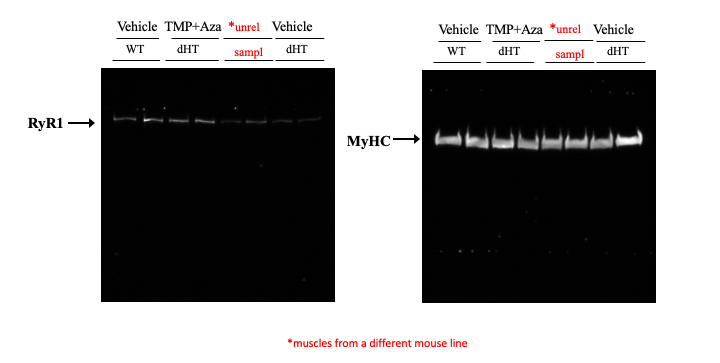

Supplement: Figure 5—source data 2. — Western blot analysis of RyR1 content in total homogenates of soleus muscle from wild type (WT) and dHT mice treated with vehicle or TMP269 + 5-Aza. Proteins were separated on a 6% SDS-PAGE, blotted overnight onto nitrocellulose. Left panel: images of blot probed with anti-RyR1 Ab used for the quantification analysis shown in the boxplot. Right panel: images of blot probed with anti-MyHC Ab for normalization. WT Vehicle: wild type mouse treated with vehicle; dHT Vehicle: dHT mouse treated with vehicle; dHT TMP + Aza; dHT mouse treated with TMP269 + 5-Aza; unrel sampl: unrelated sample (sample belonging to a mouse line of a different project). [file elife-73718-fig5-data2.zip › Figure 5B-source data 2.tiff]

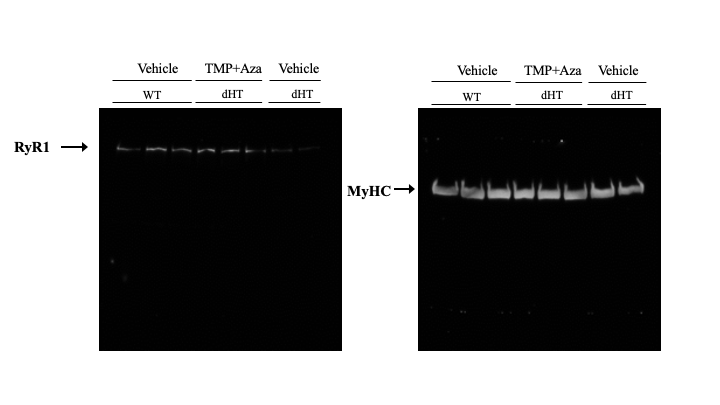

Supplement: Figure 5—source data 3. — Western blot analysis of RyR1 content in total homogenates of soleus muscle from wild type (WT) and dHT mice treated with vehicle or TMP269 + 5-Aza. Proteins were separated on a 6% SDS-PAGE, blotted overnight onto nitrocellulose. Left panel: images of blot probed with anti-RyR1 Ab used for the quantification analysis shown in the boxplot. Right panel: images of blot probed with anti-MyHC Ab for normalization. [file elife-73718-fig5-data3.zip › Figure 5B-source data 3.tiff]

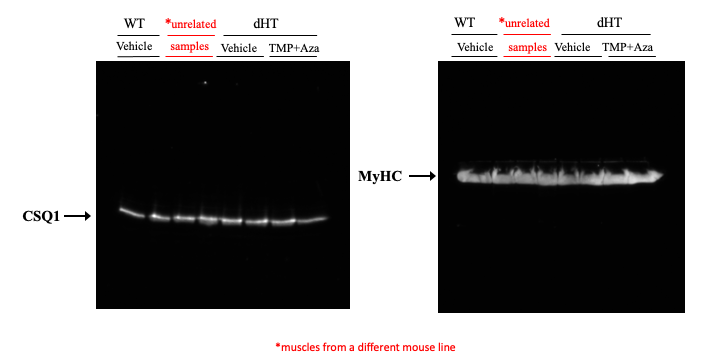

Supplement: Figure 5—source data 4. — Western blot analysis of CSQ1 content in total homogenates of soleus muscle from wild type (WT) and dHT mice treated with vehicle or TMP269 + 5-Aza. Proteins were separated on a 7.5% SDS-PAGE, blotted overnight onto nitrocellulose. Left panel: images of blot probed with anti-CSQ1 Ab used for the quantification analysis shown in the bar graph. Right panel: images of blot probed with anti-MyHC Ab for normalization. WT Vehicle: wild type mouse treated with vehicle; dHT Vehicle: dHT mouse treated with vehicle; dHT TMP + Aza; dHT mouse treated with TMP269 + 5-Aza; unrel sampl: unrelated sample (sample belonging to a mouse line of a different project). [file elife-73718-fig5-data4.zip › Figure 5C-source data 1.tif]

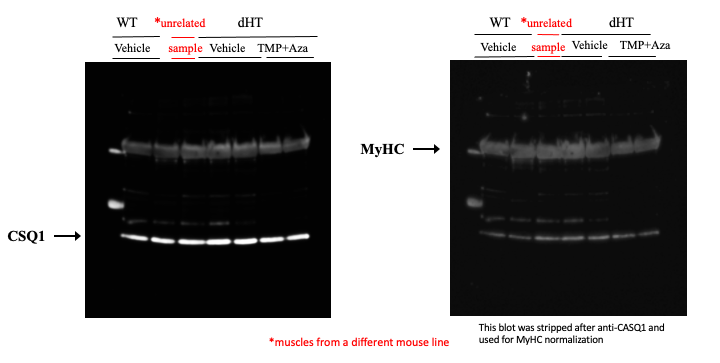

Supplement: Figure 5—source data 5. — Western blot analysis of CSQ1 content in total homogenates of soleus muscle from wild type (WT) and dHT mice treated with vehicle or TMP269 + 5-Aza. Proteins were separated on a 7.5% SDS-PAGE, blotted overnight onto nitrocellulose. Left panel: images of blot probed with anti-CSQ1 Ab used for the quantification analysis shown in the bar graph. Right panel: images of blot probed with anti-MyHC Ab for normalization. WT Vehicle: wild type mouse treated with vehicle; dHT Vehicle: dHT mouse treated with vehicle; dHT TMP + Aza; dHT mouse treated with TMP269 + 5-Aza; unrel sampl: unrelated sample (sample belonging to a mouse line of a different project). [file elife-73718-fig5-data5.zip › Figure 5C-source data 2.tif]

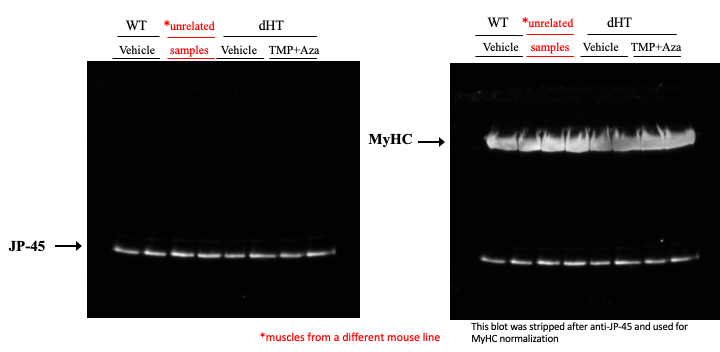

Supplement: Figure 5—source data 6. — Western blot analysis of JP-45 content in total homogenates of soleus muscle from wild type (WT) and dHT mice treated with vehicle or TMP269 + 5-Aza. Proteins were separated on a 10% SDS-PAGE, blotted overnight onto nitrocellulose. Left panel: images of blot probed with anti-JP-45 Ab used for the quantification analysis shown in the bar graph. Right panel: images of blot probed with anti-MyHC Ab for normalization. WT Vehicle: wild type mouse treated with vehicle; dHT Vehicle: dHT mouse treated with vehicle; dHT TMP + Aza; dHT mouse treated with TMP269 + 5-Aza; unrel sampl: unrelated sample (sample belonging to a mouse line of a different project). [file elife-73718-fig5-data6.zip › Figure 5C-source data 3.tif]

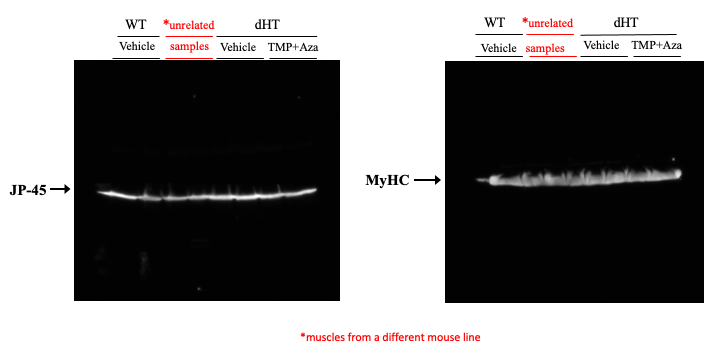

Supplement: Figure 5—source data 7. — Western blot analysis of JP-45 content in total homogenates of soleus muscle from wild type (WT) and dHT mice treated with vehicle or TMP269 + 5-Aza. Proteins were separated on a 10% SDS-PAGE, blotted overnight onto nitrocellulose. Left panel: images of blot probed with anti-JP-45 Ab used for the quantification analysis shown in the bar graph. Right panel: images of blot probed with anti-MyHC Ab for normalization. WT Vehicle: wild type mouse treated with vehicle; dHT Vehicle: dHT mouse treated with vehicle; dHT TMP + Aza; dHT mouse treated with TMP269 + 5-Aza; unrel sampl: unrelated sample (sample belonging to a mouse line of a different project). [file elife-73718-fig5-data7.zip › Figure 5C-source data 4.tif]

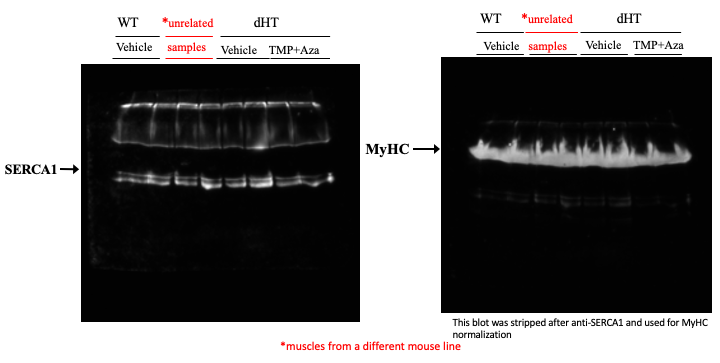

Supplement: Figure 5—source data 8. — Western blot analysis of SERCA1 content in total homogenates of soleus muscle from wild type (WT) and dHT mice treated with vehicle or TMP269 + 5-Aza. Proteins were separated on a 7.5% SDS-PAGE, blotted overnight onto nitrocellulose. Left panel: images of blot probed with anti-SERCA1 Ab used for the quantification analysis shown in the bar graph. Right panel: images of blot probed with anti-MyHC Ab for normalization. WT Vehicle: wild type mouse treated with vehicle; dHT Vehicle: dHT mouse treated with vehicle; dHT TMP + Aza; dHT mouse treated with TMP269 + 5-Aza; unrel sampl: unrelated sample (sample belonging to a mouse line of a different project). [file elife-73718-fig5-data8.zip › Figure 5C-source data 5.tif]

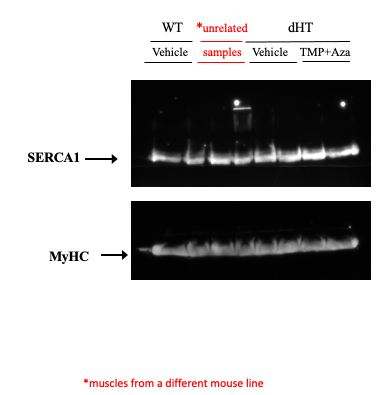

Supplement: Figure 5—source data 9. — Western blot analysis of SERCA1 content in total homogenates of soleus muscle from wild type (WT) and dHT mice treated with vehicle or TMP269 + 5-Aza. Proteins were separated on a 7.5% SDS-PAGE, blotted overnight onto nitrocellulose. Left panel: images of blot probed with anti-SERCA1 Ab used for the quantification analysis shown in the bar graph. Right panel: images of blot probed with anti-MyHC Ab for normalization. WT Vehicle: wild type mouse treated with vehicle; dHT Vehicle: dHT mouse treated with vehicle; dHT TMP + Aza; dHT mouse treated with TMP269 + 5-Aza; unrel sampl: unrelated sample (sample belonging to a mouse line of a different project). [file elife-73718-fig5-data9.zip › Figure 5C-source data 6.tif]

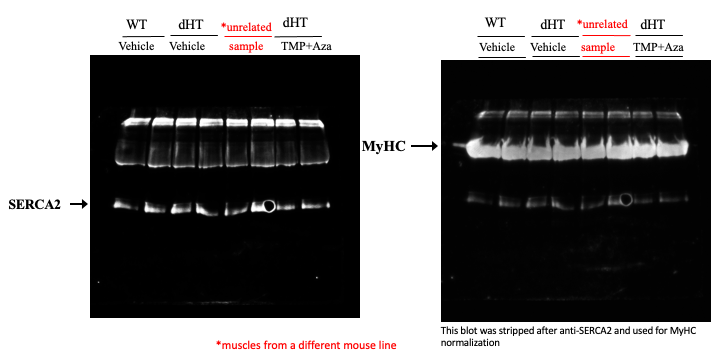

Supplement: Figure 5—source data 10. — Western blot analysis of SERCA2 content in total homogenates of soleus muscle from wild type (WT) and dHT mice treated with vehicle or TMP269 + 5-Aza. Proteins were separated on a 7.5% SDS-PAGE, blotted overnight onto nitrocellulose. Left panel: images of blot probed with anti-SERCA1 Ab used for the quantification analysis shown in the bar graph. Right panel: images of blot probed with anti-MyHC Ab for normalization. WT Vehicle: wild type mouse treated with vehicle; dHT Vehicle: dHT mouse treated with vehicle; dHT TMP + Aza; dHT mouse treated with TMP269 + 5-Aza; unrel sampl: unrelated sample (sample belonging to a mouse line of a different project). [file elife-73718-fig5-data10.zip › Figure 5C-source data 7.tif]

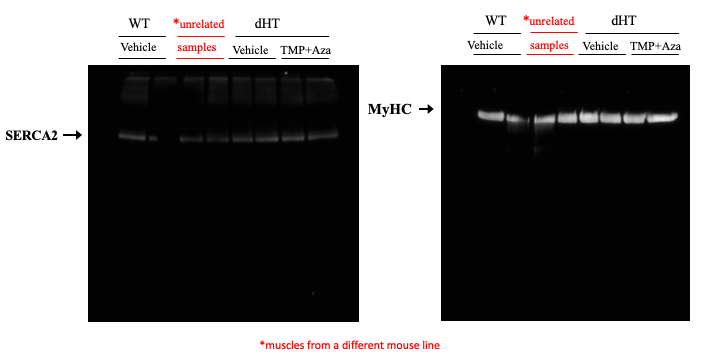

Supplement: Figure 5—source data 11. — Western blot analysis of SERCA2 content in total homogenates of soleus muscle from wild type (WT) and dHT mice treated with vehicle or TMP269 + 5-Aza. Proteins were separated on a 7.5% SDS-PAGE, blotted overnight onto nitrocellulose. Left panel: images of blot probed with anti-SERCA1 Ab used for the quantification analysis shown in the bar graph. Right panel: images of blot probed with anti-MyHC Ab for normalization. WT Vehicle: wild type mouse treated with vehicle; dHT Vehicle: dHT mouse treated with vehicle; dHT TMP + Aza; dHT mouse treated with TMP269 + 5-Aza; unrel sampl: unrelated sample (sample belonging to a mouse line of a different project). [file elife-73718-fig5-data11.zip › Figure 5C-source data 8.tif]

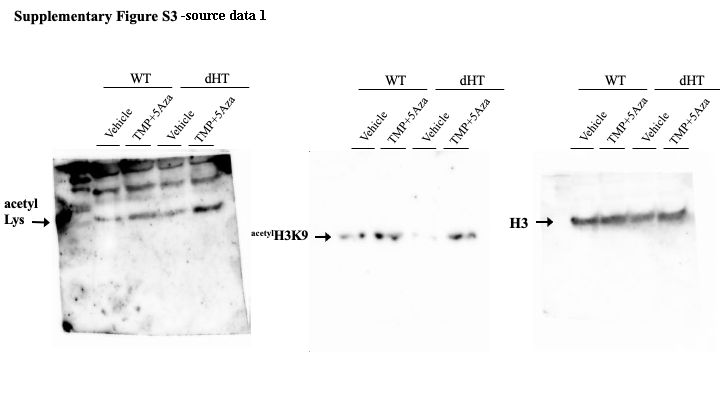

Supplement: Appendix 2—figure 2—source data 1. [file elife-73718-app2-fig2-data1.tiff]

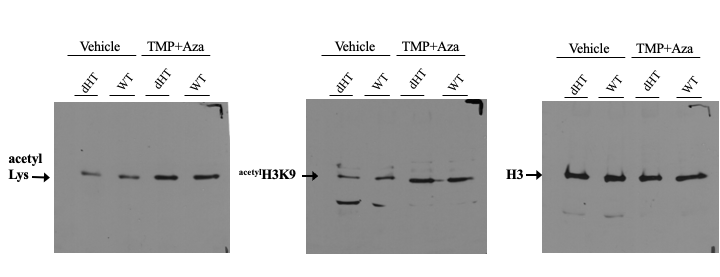

Supplement: Appendix 2—figure 2—source data 2. — Wild type (WT) and dHT mice received a daily injection vehicle or 25 mg/kg TMP269+0.05 mg/kg 5-Aza. After 15 weeks of treatment, approximately 200 flexor digitorum brevis (FDBs) fibers per mouse were isolated and resuspended in cracking buffer. Protein extracts were loaded onto a Tris-Tricine gel, blotted onto nitrocellulose, and probed as indicated, with anti-acetyl-Lys antibodies, anti-H3K9acetyl antibodies, and anti-H3 antibodies (loading control). The three panels show the immunoreactive bands (arrows) that were used for the Boxplot analysis of Appendix 2—figure 2. The immunoreactivity of the H3K9acetyl positive band obtained in muscles from vehicle-treated WT mice was set to 100%. [file elife-73718-app2-fig2-data2.zip › Appendix 2-figure 2 source data 5.tif]

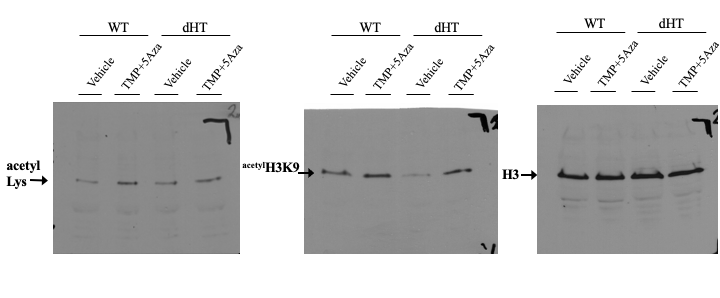

Supplement: Appendix 2—figure 2—source data 3. — Wild type (WT) and dHT mice received a daily injection vehicle or 25 mg/kg TMP269+0.05 mg/kg 5-Aza. After 15 weeks of treatment, approximately 200 flexor digitorum brevis (FDBs) fibers per mouse were isolated and resuspended in cracking buffer. Protein extracts were loaded onto a Tris-Tricine gel, blotted onto nitrocellulose, and probed as indicated, with anti-acetyl-Lys antibodies, anti-H3K9acetyl antibodies, and anti-H3 antibodies (loading control). The three panels show the immunoreactive bands (arrows) that were used for the Boxplot analysis of Appendix 2—figure 2. The immunoreactivity of the H3K9acetyl positive band obtained in muscles from vehicle-treated WT mice was set to 100%. [file elife-73718-app2-fig2-data3.zip › bbb3b35d-9b52-4c60-9b63-dd7d14b2607d.tif]

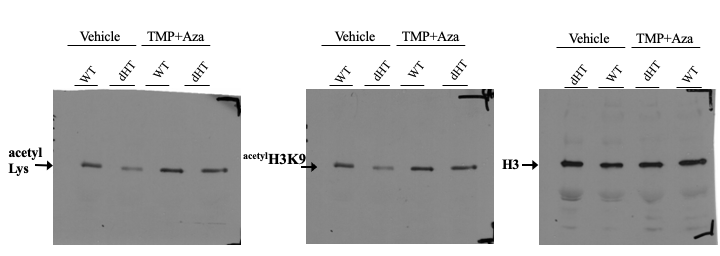

Supplement: Appendix 2—figure 2—source data 4. — Wild type (WT) and dHT mice received a daily injection vehicle or 25 mg/kg TMP269+0.05 mg/kg 5-Aza. After 15 weeks of treatment, approximately 200 flexor digitorum brevis (FDBs) fibers per mouse were isolated and resuspended in cracking buffer. Protein extracts were loaded onto a Tris-Tricine gel, blotted onto nitrocellulose, and probed as indicated, with anti-acetyl-Lys antibodies, anti-H3K9acetyl antibodies, and anti-H3 antibodies (loading control). The three panels show the immunoreactive bands (arrows) that were used for the Boxplot analysis of Appendix 2—figure 2. The immunoreactivity of the H3K9acetyl positive band obtained in muscles from vehicle-treated WT mice was set to 100%. [file elife-73718-app2-fig2-data4.zip › Appendix 2-figure 2 source data 4.tif]
