# Supplementary material for: Improvement of muscle strength in a mouse model for congenital myopathy treated with HDAC and DNA methyltransferase inhibitors
Source: eLife. 2022 Mar 3;11:e73718. doi: 10.7554/eLife.73718 (PMC8956288; doi:10.7554/eLife.73718)
Supplement: Supplementary file 1. [file elife-73718-supp1.docx]

**Supplementary File 1**. List of hypomethylated protein-encoding genes in soleus muscles from dHT mice treated for 15 weeks with TMP269+5-Aza drug versus vehicle treated dHT mice.

| **Gene name** | **Gene name** | **Gene name** | **Gene name** | **Gene name** |
| --- | --- | --- | --- | --- |
| *Atp1b2* | *Sash3* | *Dnase1L1* | *Atrx* | *Tenm1* |
| *Itgb8* | *Utp14a* | *Slc10a3* | *Magt1* | *Ocrl* |
| *Prkcd* | *Elf4* | *Ubl4a* | *Atp7a* | *Phf8* |
| *Gjb6* | *Aifm1* | *Ikbkg* | *Taf9b* | *Huwe1* |
| *Pou6f1* | *Zfp280c* | *Tbl1x* | *Brwd3* | *Smc1a* |
| *Cdc25c* | *Enox2* | *Prkx* | *Chm* | *Klf8* |
| *Adnp2* | *Firre* | *Prrg1* | *Diaph2* | *Acot9* |
| *Uty* | *Mbnl3* | *Dmd* | *Pcdh19* | *Sms* |
| *Kcnt1* | *Hs6st2* | *Il1rapl1* | *Cstf2* | *Cnksr2* |
| *Kcnd3* | *Usp26* | *Pola1* | *Arl13a* | *Bclaf3* |
| *Prkag2* | *Gpc4* | *AL646049.1* | *Nox1* | *Sh3kbp1* |
| *Asb4* | *Plac1* | *Heph* | *Cenpi* | *Map3k15* |
| *Cav2* | *Fam122b* | *Ophn1* | *Drp2* | *Adgrg2* |
| *Fbln2* | *Fam122c* | *Stard8* | *Armcx4* | *Phka2* |
| *Rgma* | *Zfp36l3* | *Efnb1* | *Gprasp1* | *Cdkl5* |
| *Bckdk* | *Ints6l* | *Eda* | *Bhlhb9* | *Gja6* |
| *Ddx3y* | *Mmgt1* | *Awat2* | *Tbc1d8b* | *Nhs* |
| *Usp9y* | *Slc9a6* | *Otud6a* | *Rbm41* | *Piga* |
| *Shroom4* | *Fhl1* | *Igbp1* | *Tex13b* | *Gpm6b* |
| *Clcn5* | *Arhgef6* | *Kif4* | *Nxt2* | *Ofd1* |
| *Usp27x* | *Fgf13* | *Dlg3* | *Acsl4* | *Mid1* |
| *Foxp3* | *Tmem185a* | *Snx12* | *Tmem164* |  |
| *Gpkow* | *Mamld1* | *Foxo4* | *Ammecr1* |  |
| *Ccdc120* | *Gabre* | *Il2rg* | *Pak3* |  |
| *Otud5* | *Zfp275* | *Zmym3* | *Lhfpl1* |  |
| *Pcsk1n* | *Atp2b3* | *Ogt* | *Lrch2* |  |
| *Gata1* | *Dusp9* | *Nhsl2* | *Alas2* |  |
| *Fthl17f* | *Pnck* | *Rps4x* | *Apex2* |  |
| *Rpgr* | *Slc6a8* | *Cited1* | *Tro* |  |
| *Tspan7* | *Renbp* | *Dmrtc1b* | *Gnl3l* |  |
| *Bcor* | *Mecp2* | *Zdhhc15* | *Fam120c* |  |
| *Med14* | *Rbm10* | *Klhl13* | *Upf3b* |  |
| *Nyx* | *Cdk16* | *Dock11* | *Tmem255a* |  |
| *Cask* | *Usp11* | *Il13ra1* | *Lamp2* |  |
| *Fundc1* | *Araf* | *Lonrf3* | *Cul4b* |  |
| *Slc9a7* | *Elk1* | *Septin6* | *Gria3* |  |
