# Supplementary material for: Improvement of muscle strength in a mouse model for congenital myopathy treated with HDAC and DNA methyltransferase inhibitors
Source: eLife. 2022 Mar 3;11:e73718. doi: 10.7554/eLife.73718 (PMC8956288; doi:10.7554/eLife.73718)
Supplement: Supplementary file 2. [file elife-73718-supp2.docx]

**Supplementary File 2:** Specific force of EDL and soleus muscle from WT and dHT mice treated with vehicle or TMP269+5-Aza for 15 weeks. Muscles were stimulated with a single twitch or tetanic stimulation (EDL: 150 Hz, 400 ms duration; soleus 120 Hz, 400 ms duration). Values are expressed as specific force (mN/mm^2^) *p <0.05 **p<0.01 dHT vs WT; ¶ p<0.05 dHT vehicle vs dHT TMP269+5-Aza (ANOVA followed by the Bonferroni post hoc test).

(ANOVA followed by the Bonferroni post hoc test).

|  |  | **EDL** | | **soleus** | |
| --- | --- | --- | --- | --- | --- |
| **Genotype** | **Treatment** | **Twitch**  (mean±SD) | **Tetanus**  **150 Hz**  (mean±SD) | **Twitch**  (mean±SD) | **Tetanus**  **120 Hz**  (mean±SD) |
| WT | Vehicle (n=8) | 171.24±29.32 | 452.96±89.58 | 96.58±25.78 | 315.86±56.96 |
| dHT | Vehicle  (n=10)  (P value) | **64.92±13.93  (P=0.0015) | *373.76±73.16  (P=0.036) | *67.55±11.26  (P=0.040) | *276.29±40.04  (P=0.043) |
|  | TMP269 + 5Aza  (n=13)  (P value) | *74.37±41.30  (P=0.012) | *379.96±87.87  (P=0.044) | *^¶^84.61±14.06  (*P=0.048)  (¶P=0.047) | *^¶^334.78±65.74  (*P=0.047)  (¶P=0.048) |
|  |  |  |  |  |  |
