# Supplementary material for: Improvement of muscle strength in a mouse model for congenital myopathy treated with HDAC and DNA methyltransferase inhibitors
Source: eLife. 2022 Mar 3;11:e73718. doi: 10.7554/eLife.73718 (PMC8956288; doi:10.7554/eLife.73718)
Supplement: Supplementary file 3. [file elife-73718-supp3.docx]

**Supplementary File 3.** Fiber type composition of soleus muscles from mice treated for 15 weeks with vehicle or TMP269+5-Aza.

|  | **MyHC I/total**  (Ratio ±SEM) | **MyHC IIa/total**  (Ratio ±SEM) | **MyHC IIx/total**  (Ratio ±SEM) | **Total N° fibers counted** |
| --- | --- | --- | --- | --- |
|  |  |  |  |  |
| **WT vehicle** | 0.31± 0.005 | 0.50±0.019 | 0.16±0.008 | 2881 |
| **dHT vehicle** | 0.33±0.02 | 0.53±0.02 | 0.13±0.008 | 2642 |
| **dHT treated** | 0.27±0.0059 | 0.56±0.015 | 0.15±0.01 | 2983 |
