# Supplementary material for: Improvement of muscle strength in a mouse model for congenital myopathy treated with HDAC and DNA methyltransferase inhibitors
Source: eLife. 2022 Mar 3;11:e73718. doi: 10.7554/eLife.73718 (PMC8956288; doi:10.7554/eLife.73718)
Supplement: Supplementary file 4. [file elife-73718-supp4.docx]

**Supplementary File 4:** Analysis of electrically evoked calcium transients in single FDB muscle fibers isolated from WT and dHT littermates, treated with vehicle or TMP269+5-Aza (25 mg/Kg) for 15 weeks. *p<0.05 dHT vs WT;^¶^ p<0.05 dHT vehicle vs dHT TMP269+5-Aza (ANOVA followed by the Bonferroni post hoc test).

|  | **Treatment** | **Number of mice/N° of fibers analyzed** | **∆F/F**  **(mean±S.D.)** | **TTP msec**  **(mean±S.D.)** | **HTTP ms**  **(mean±S.D.)** | **HRT ms**  **(mean±S.D.)** |  |
| --- | --- | --- | --- | --- | --- | --- | --- |
| **Twitch** | WT | Vehicle  (NMP/PEG) | 4  (n=91) | 1.38±0.30 | 1.29±0.90 | 0.80±0.27 | 2.06±1.75 |
|  | dHT | Vehicle  (NMP/PEG)  (P value) | 6  (n=110) | *0.98±0.22  (P=0.038) | 1.29±0.65 | 0.78±0.27 | 2.07±1.20 |
|  | dHT | TMP269 +5Aza  (P value) | 5  (n=155) | ^¶^1.21±0.28  (P=0.043) | 1.29±0.30 | 0.81±0.22 | 1.94±1.67 |
| **Tetanic** | WT | Vehicle  (NMP/PEG) | 4  (n=63) | 1.62±0.21 |  |  |  |
|  | dHT | Vehicle  (NMP/PEG)  (P value) | 6  (n=92) | *1.22±0.22  (P=0.039) |  |  |  |
|  | dHT | TMP269 +5Aza  (P value) | 5  (n=78) | ^¶^1.42±0.23  (P=0.042) |  |  |  |
