# Supplementary material for: Improvement of muscle strength in a mouse model for congenital myopathy treated with HDAC and DNA methyltransferase inhibitors
Source: eLife. 2022 Mar 3;11:e73718. doi: 10.7554/eLife.73718 (PMC8956288; doi:10.7554/eLife.73718)
Supplement: Supplementary file 5. [file elife-73718-supp5.docx]

**Supplementary File 5:** Sequence of primers used and gene targets

| **Primer details** | | **Primer sequence** | |
| --- | --- | --- | --- |
|  | | **Forward** | **Reverse** |
| Ex36 *Ryr1* | Genotyping | TGCTGGCTTCAGAGTGAT CG | CGAGGGAAGTTGAGGTTGGG |
| Ex91 *Ryr1* | Genotyping | GAGATGTTCGTGAGTTTCTGCGAGG | TGAGGGTTGTTCTTGGTGTATTTGG |
| *Ryr1* | qPCR | CGCCAAAACGGAGAGAAAGTC | TTGATGGTGGTCGTGTTCCC |
| *Cacna1s* | qPCR | TCAGCATCGTGGAATGGAAAC | GTTCAGAGTGTTGTTGTC |
| *Hdac4* | qPCR | CACTGCATTTCCAGCGATCC | AAGACGGGGTGGTTGTAGGA |
| *Gapdh* | qPCR | CTGCACCACCAACTGCTTAGC | GGCATGGACTGTGGTCATGAG |
