# Supplementary material for: Improvement of muscle strength in a mouse model for congenital myopathy treated with HDAC and DNA methyltransferase inhibitors
Source: eLife. 2022 Mar 3;11:e73718. doi: 10.7554/eLife.73718 (PMC8956288; doi:10.7554/eLife.73718)
Supplement: Supplementary file 6. [file elife-73718-supp6.docx]

**Supplementary File 6:** List of antibodies and suppliers

| **Target** | **Supplier** | **Catalog number** |
| --- | --- | --- |
| Acetyl Lysine | Abcam | Ab21623 |
| Alexa Fluor 568 | ThermoFisher Scientific | 21124 |
| Alexa Fluor 488 | ThermoFisher Scientific | 21042 |
| Calsequestrin 1 | Sigma | C0742 |
| Cav1.1 | DSHB | IIC12D4 |
| Histone H3 | Abcam | Ab1791 |
| Histone H3 (acetyl K9) | Abcam | Ab10812 |
| JP-45 | Lab housemade | Zorzato et al., 2000 |
| Laminin | Sigma | L9393 |
| MyHC | Santa Cruz | Sc-376157 |
| MyHC I | DSHB | BA-D5 |
| MyHC-IIa | DSHB | SC-71 |
| MyHC IIb | DSHB | BF-F3 |
| RyR1 | Cell Signaling | D4E1 |
| SERCA1 | Santa Cruz | sc-8093 |
| SERCA2 | Santa Cruz | sc-8095 |
