# Supplementary material for: Improvement of muscle strength in a mouse model for congenital myopathy treated with HDAC and DNA methyltransferase inhibitors
Source: eLife. 2022 Mar 3;11:e73718. doi: 10.7554/eLife.73718 (PMC8956288; doi:10.7554/eLife.73718)
Supplement: Transparent reporting form [file elife-73718-transrepform1.pdf]

eLIFE
1st Floor
24 Hills Road
Cambridge CB2 1JP, UK
P 01223 855340
W elifesciences.org
T @elife

eLife's transparent reporting form

We encourage authors to provide detailed information *within their submission* to facilitate the interpretation and replication of experiments. Authors can upload supporting documentation to indicate the use of appropriate reporting guidelines for health-related research (see EQUATOR Network), life science research (see the BioSharing Information Resource), or the ARRIVE guidelines for reporting work involving animal research. Where applicable, authors should refer to any relevant reporting standards documents in this form.

If you have any questions, please consult our Journal Policies and/or contact us: editorial@elifesciences.org.

Sample-size estimation

- You should state whether an appropriate sample size was computed when the study was being designed
- You should state the statistical method of sample size computation and any required assumptions
- If no explicit power analysis was used, you should describe how you decided what sample (replicate) size (number) to use

Please outline where this information can be found within the submission (e.g., sections or figure legends), or explain why this information doesn't apply to your submission:

- We had no idea a priori as to of the differences to be expected between control and drug treated animals, therefore no explicit power of analysis was used to estimate samples size.
- To define the number of animals used for the *in vivo* and *in vitro* assays we followed the 3Rs guidelines, as recommended by the Kantonal Veterinary Authorities.
- Mice were divided based on their genotypes and then randomly assigned to the different treatment groups.
- For statistical analysis a minimum of 3 mice per group were used. For differences of approx. 20% and whenever possible, the number of mice included in each group was up to 11. For larger differences, the number of mice included in the experiments was reduced. For EM studies, soleus muscles from 2 vehicle-treated and 2 drug treated mice were analysed.
- Sample size is indicated in the text, methods section, results section and figure legends. In particular we indicate how many mice per group were utilized in each experiment and, when appropriate, how many fibers from a total of x different mice, were analysed.

Replicates

- You should report how often each experiment was performed
- You should include a definition of biological versus technical replication
- The data obtained should be provided and sufficient information should be provided to indicate the number of independent biological and/or technical replicates
- If you encountered any outliers, you should describe how these were handled
- Criteria for exclusion/inclusion of data should be clearly stated

- High-throughput sequence data should be uploaded before submission, with a private link for reviewers provided (these are available from both GEO and ArrayExpress)

Please outline where this information can be found within the submission (e.g., sections or figure legends), or explain why this information doesn't apply to your submission:

The information concerning replicates can be found in the Methods section.
All experiments show data on biological replicates (i.e. individual mice); technical replicates are present in figure 4 where we performed calcium measurements on single FDB fibers (n ranging from 63-155) isolated from 4-6 mice. Because of fiber type heterogeneity of FDB fibers, we performed calcium measurments in a large number of single fibers isolated per mouse. Detailed information is given in Table S4. For the results of qPCR experiments shown in Figure 5A, each symbol represents one biological sample and the reaction was performed in duplicate and averaged. This information in included in the Methods section.
No mice were excluded form the data points (i.e we did not remove any outliers).

Statistical reporting

- Statistical analysis methods should be described and justified
- Raw data should be presented in figures whenever informative to do so (typically when N per group is less than 10)
- For each experiment, you should identify the statistical tests used, exact values of N, definitions of center, methods of multiple test correction, and dispersion and precision measures (e.g., mean, median, SD, SEM, confidence intervals; and, for the major substantive results, a measure of effect size (e.g., Pearson's r, Cohen's d)
- Report exact p-values wherever possible alongside the summary statistics and 95% confidence intervals. These should be reported for all key questions and not only when the p-value is less than 0.05.

Please outline where this information can be found within the submission (e.g., sections or figure legends), or explain why this information doesn't apply to your submission:

- Statistical analysis methods are reported in the Statistical analysis section at the end of the Methods section.
- The statistical method that was used is also indicated in the figure legend accompanying each figure, in the results section and in the Table footnotes.
- Whenever data are not presented as box plot, the reported values indicate the mean ±SD value. Except for Figure 3 and Tables a and 2 where the mean± SEM are given.
- The raw data is shown in the Figures and Tables.
- Exact P values are given in Supplementary Figure S2, in Supplementary Tables S2 and S4 and in the text.

(For large datasets, or papers with a very large number of statistical tests, you may upload a single table file with tests, Ns, etc., with reference to sections in the manuscript.)

Group allocation

- Indicate how samples were allocated into experimental groups (in the case of clinical studies, please specify allocation to treatment method); if randomization was used, please also state if restricted randomization was applied
- Indicate if masking was used during group allocation, data collection and/or data analysis

Please outline where this information can be found within the submission (e.g., sections or figure legends), or explain why this information doesn't apply to your submission:

- For group allocation, mice were genotyped and randomly assigned to vehicle or drug treatment.
- For *ex vivo* muscle force determination and grip strength assessment the experimenter was blinded as to the mouse genotype and drug treatment. This information is included in the text (results section).

Additional data files ("source data")

- We encourage you to upload relevant additional data files, such as numerical data that are represented as a graph in a figure, or as a summary table
- Where provided, these should be in the most useful format, and they can be uploaded as "Source data" files linked to a main figure or table
- Include model definition files including the full list of parameters used
- Include code used for data analysis (e.g., R, MatLab)
- Avoid stating that data files are "available upon request"

Please indicate the figures or tables for which source data files have been provided:

The numerical data that are represented in Figures 2, 3 and 4 are given in Supplementary Tables S2, S3 and S4.

eLife Sciences Publications, Ltd is a limited liability non-profit non-stock corporation incorporated in the State of Delaware, USA, with company number 5030732, and is registered in the UK with company number FC030576 and branch number BR015634 at the address 1st Floor, 24 Hills Road, Cambridge CB2 1JP | August 2014
